# Developing and evaluating online COVID-centric advance care planning training and information resources for nursing staff and family members in nursing homes: the necessary discussions study protocol

**DOI:** 10.1186/s12877-021-02398-1

**Published:** 2021-08-09

**Authors:** Andrew Harding, Nancy Preston, Julie Doherty, Emily Cousins, Sandra Varey, Adrienne McCann, Karen Harrison Dening, Anne Finucane, Gillian Carter, Gary Mitchell, Kevin Brazil

**Affiliations:** 1 Division of Health Research, Faculty of Health and Medicine, Lancaster University, Lancaster, UK; 2 School of Nursing and Midwifery, Centre for Evidence and Social Innovation, Queen’s University Belfast, Belfast, UK; 3 School of Nursing and Midwifery, Faculty of Health and Life Sciences, De Montfort University, Leicester & Dementia UK, London, UK; 4 Clinical Psychology, University of Edinburgh, Edinburgh & Marie Curie Hospice Edinburgh, Edinburgh, UK

**Keywords:** Advance care planning, COVID-19, Coronavirus, Nursing homes, Training, Information, E-learning, Online training

## Abstract

**Background:**

Nursing home residents are typically older adults with high levels of chronic illness and impairment. As such, they are particularly susceptible to severe complications and mortality from COVID-19. Since all nursing home residents are at increased risk, nursing home care staff need to know what residents would want to happen should they become infected with COVID-19. This study aims to develop and evaluate advance care planning (ACP) COVID-centric online training and information resources for nursing home staff and family members of residents, to improve care at the end of life during a COVID-19 outbreak. Based on the findings we will develop implementation guidelines for nursing homes to ensure wider impact and application during the pandemic and beyond.

**Methods:**

The content of the training and information resources will be based on a rapid review of literature and guidance on ACP in the context of COVID-19 and consultation with the study expert reference group. An integrated communications company will then work alongside the research team to design the online training and information resources. To evaluate the resources, we will employ a multiple case study design where a nursing home (defined as an institutional setting in which nursing care is provided to older adults on-site 24 h a day) will be the unit of analysis or ‘case’. The RE-AIM (reach, effectiveness, adoption, implementation, maintenance) framework will guide the evaluation of implementation of the training and information resources. We will recruit and interview staff and family members from between 6 and 9 nursing homes across Northern Ireland, England and Scotland and gather quantitative data from a feedback survey included in the training and information resources.

**Discussion:**

The Necessary Discussions study is very timely given the challenging experiences of nursing homes, their staff, residents and their family members during the COVID-19 pandemic. It meets a key need and addresses an important gap in research and practice. The training and information resources will be ‘COVID-centric’, but they will also have a longstanding relevance for future ACP practice in UK care homes.

**Trial registration:**

ISRCTN registry (ID 18003630) on 19.05.21

## Background

Nursing home residents are typically older adults with high levels of chronic illness and impairment. As such, they are particularly susceptible to severe complications and mortality from COVID-19 [1]. Since all nursing home residents are at substantial risk, nursing home staff need to know what residents would want to happen should they become infected with COVID-19. One way of ensuring that care reflects the wishes and preferences of residents, is to develop an advance care plan. Although it is regarded as good practice for advance care plans to be in place at the earliest possible opportunity upon admission to a nursing home, the COVID-19 pandemic and the sudden manner of illness and rapid deterioration of nursing home residents, and in some cases death, has amplified the need for documented advance care plans [2, 3].

Through a consensus study, Sudore and colleagues provide the following definition: “Advance care planning is a process that supports adults at any age or stage of health in understanding and sharing their personal values, life goals, and preferences regarding future medical care. The goal of advance care planning (henceforth referred to as ACP) is to help ensure that people receive medical care that is consistent with their values, goals and preferences during serious and chronic illness. or many people, this process may include choosing and preparing another trusted person or persons to make medical decisions in the event the person can no longer make his or her own decisions.” [4]. More broadly, ACP is viewed as an on-going conversation which should involve the provision of information [5] to patients’ / residents’ and family members’ “… as a means of navigating the uncertainties at the end of life by identifying and supporting patients’ needs and preferences for care.” [6].

It is good practice for ACP to have several documented outcomes, including clarification of patient’s preferences, values and aims for future medical treatments and care; the identification of the refusal of specific future treatments should the patient or resident later become unable to communicate; and the appointment of a person to take decisions on the patient’s behalf if they subsequently lose decisional capacity [6, 7].

ACP conversations and processes are regarded as difficult and often complex for both nursing home staff and family members [3–6]. However, given the emergence of COVID-19 as a global pandemic, as classified by the World Health Organisation on the 11th March 2020, ACP practice and its potential for ensuring high quality person-centred care at the end of life, has arguably become more important than ever. It is vitally important that both nursing home staff and family members feel competent and confident to engage in ACP conversations. The provision of training and information resources are means of empowering these groups to have these difficult but ‘necessary discussions’. However, critical gaps have been highlighted in research, policy and practice around ACP tools, training and information resources, which are exacerbated within the context of COVID-19.

In a broader nursing home context, the effectiveness of staff training [8] and quality and accessibility of information resources for family members [9–11] have both been frequently cited as being underdeveloped fields of research and practice. Furthermore, with respect to COVID-19, a recent review published by The Centre for Evidence Based Medicine at the University of Oxford on ACP in the community [3] found that 1) there often exists a lack of awareness among key stakeholders about ACP; 2) there is a need to develop information and training resources – particularly online – to support ACP practice; 3) there is a paucity of research on ACP in a COVID-19 context.

The aim of the ‘Necessary Discussions’ study is to address these aforementioned gaps and develop and evaluate ACP COVID-centric online training and information resources, alongside implementation guidelines for nursing homes to improve care at the end of life during a COVID-19 outbreak. Moreover, the study will:

1. Develop an online ACP COVID-centric training resource (a website) for nursing home staff including conversational prompts to aid conversations.
2. Prepare an online ACP COVID-centric information resource for family members.
3. Evaluate the training and information resources and identify the facilitators and barriers to implementing these resources in a nursing home context.
4. Produce implementation guidelines for online ACP COVID-centric resources that will facilitate widespread use within UK nursing homes.
5. Identify strategic partnerships that will facilitate the integration of the ACP online resources into nursing home practice.

## Methods/design

### Design

The study will employ a multiple mixed methods case study design where a nursing home will be the unit of analysis or ‘case’ [12]. Case study is an approach that is widely adopted in nursing and care home research [13], as well as related areas such as palliative care [14, 15]. Central to a case study approach is to triangulate multiple methods and sources of evidence to explore phenomena that is both complex and context-dependant (Yin, 2009). A prospective case study design will allow us to understand the implementation process, and barriers and facilitators for the educational and information resources. The RE-AIM (reach, effectiveness, adoption, implementation, maintenance) framework will guide the evaluation of the educational and information resources [16]. There are four key phases to the study:

1. Establishment of an Expert Reference Group (ERG) and International Observer Panel (IOP)
2. Rapid review of ACP resources
3. Development of the educational and information resources (website)
4. Evaluation of the ACP training and information resources and development of the implementation guidance

### Phase 1

#### Establishment of an expert reference group and international observer panel

The Expert Reference Group (ERG) will include up to 14 individuals. The group will include representation from nursing home managers, nursing home direct care providers, public involvement representation including family members, knowledge expert on ACP, third sector organizations (e.g., Age UK, Marie Curie, Dementia UK), and a healthcare provider with COVID-19 care experience. The ERG will provide advice on all appropriate aspects of the project, review project progress and outputs, adherence to the protocol, and act as a network based on the project objectives concerning dissemination. The ERG will meet as a group to discuss intervention development at three milestone points: 1) at the project launch; 2) completion of the content for the ACP online resources; 3) reviewing evaluation results. Outside of these meetings ERG members will be consulted on an individual basis on study matters where necessary. All communication will be conducted on MS Teams (GDPR compliant) or via telephone.

Recognising COVID-19 as a global pandemic, an international observer panel (IOP) of experts in palliative, nursing home care will be established. Drawn from Italy, Czech Republic, Netherlands, Republic of Ireland and Canada this panel will monitor project activities with the view of applying outputs in their home countries and for wider dissemination purposes.

### Phase 2

#### Rapid review of ACP resources

A review of currently available UK ACP resources that focus on COVID-19 will be conducted. ACP resources will be included from government and professional association websites and personal communications. We will use ACP resources that are known to the research team, put a ‘call out’ on social media platforms and run guideline specific searches on the Turning Research into Practice (TRIP) database and Google. A review and synthesis of relevant material will contribute to the development of the online ACP COVID- centric training resource for nursing home staff and family members by identifying existing content, gaps in current resources specific to a UK context.

### Phase 3

#### Development of online training and information resources

Staff training and information resources for family members will be developed using findings from phase 2. An integrated communications company will be commissioned to develop a website to present the resources for each group. The company specialise in digital resources and have worked with a vast range of public and private sector clients.

It is anticipated that the following content will be developed: expert interviews giving guidance on topics and communication tips; interviews with staff about real life experiences, enabling peer to peer learning; resource planning tips, including handling technology; infographics highlighting key messages and practical techniques. It is anticipated that the training resource for nursing home staff will cover the following themes: an introduction to advance care planning, including benefits and misconceptions; advance care planning in the context of COVID-19, including emergency care planning; how to complete an advance care plan during COVID-19, including hosting a family meeting online; recording and sharing advance care plans during COVID-19, including identifying stakeholders; tips and guidance for having advance care planning conversations; self-care during COVID-19. Communication tips will be embedded in each theme. Due to infection control protocols during a COVID-19 outbreak conversation prompts for ACP conversations will be developed in anticipation that nursing staff communication with family members will frequently be via telephone or video only. It is anticipated that the online ACP information resource for family members will include information on key terms used in ACP; review of ACP, its purpose, contents who is involved and ways to take part in ACP conversations during COVID-19.

The research team and ERG will review and provide feedback on the training and information resources prior to developing the online platform. The language used will be simple and informative to ensure it is user friendly. Future considerations will include the use of subtitles that will allow for inclusiveness of those with sight or hearing impairments and people who do not speak English.

### Phase 4

#### Evaluation of the ACP online intervention

The study will employ a case study approach where a nursing home will be the unit of analysis or ‘case’. Participants at each nursing home (care staff and family members) will be asked to complete the training (the intervention) and provide feedback (the evaluation). A prospective case study design will allow us to understand the implementation process for the intervention (1, 2). The team have established relationships with many nursing home providers who have indicated their willingness to participate in what they see as vital research. The NIHR initiative Enabling Research in Care Homes (ENRICH) network will also be engaged, if needed, in recruiting nursing homes.

The RE-AIM framework will guide the evaluation [16]. A major feature of RE-AIM is that it shifts the focus from randomized efficacy trials to longer-term effectiveness in a real- world setting. The dimensions of the framework are as follows:

- Reach, the proportion and representativeness of identified nursing home staff, family members and residents who access the online training resource and review resource features
- Effectiveness, the impact the ACP online intervention has on staff and family members knowledge and attitudes towards the intervention (content, aesthetic properties, usability) and ACP
- Adoption, assess nursing home staff and family member acceptability of the training
- Implementation, assessment of the fidelity of implementing the protocol
- Maintenance, determine whether nursing home managers and staff wish to continue the intervention

#### Settings, participants and data collection

Sampling will occur at three levels in this project: (1) across geographic locations; (2) selection of multiple quantitative and qualitative data sources within each nursing home; and (3) ensuring a spread of staff and family member characteristics. The study aims to recruit up to 9 nursing homes across Northern Ireland (*n* = 3), Scotland (*n =* 3) and England (*n =* 3). We aim to recruit within each site (and in total) (Table 1):

- 1 nursing home manager (*n =* 9)
- 4 nursing home staff per site (*n =* approximately 36)
- 6 family members relating to 6 residents (*n =* approximately 54)

**Table 1:** **Table 1** Phases, data collection, methods and themes

Phase	Data collection methods and themes
**1. Environmental Scan**	**Interviews with nursing home manager** ***(n = 1)*** **Key themes:** Current practice; Impressions of the proposed COVID; ACP intervention; Potential barriers and facilitators towards implementation. **Nursing home profile questionnaire** ***(administered as part of interview with nursing home manager)*** **Key themes:** Resident and facility profile (including COVID outbreaks); Care home policies and procedures; Staff profile; Staff training; Use of technology; Contact with health and social care professionals; Response to COVID-19. **Interviews with nursing homes staff** ***(n = up to 4 registered nurses who undertake training)*** **Key themes:** Demographics**;** Current practice; Experience (including confidence & concerns about completing ACP in the context of COVID-19); Communication & technology; The role of family members; Impressions of the COVID-19 online training; Potential barriers & facilitators. **Interviews with family carers** ***(up to n = 6)*** **Key themes:** Background information/Demographics; Use of technology and current communication approach with nursing home/relative; Information needs
**3. Follow up data collection** Completed 1–2 weeks following delivery of the online training by participants recruited during the environmental scan	**Interviews with nursing home managers, staff, family carers & the external facilitator** ***(n = see above)*** **Key themes. Family carers:** Satisfaction; Acceptability; Impact on attitudes; knowledge & behaviour; Technology & communication**. Nursing home manager/staff**: Satisfaction; Acceptability; Impact on attitudes, knowledge & behaviour; Communication & technology; Implementation. **External facilitator**: Satisfaction; Acceptability; Training & sustainability. **Feedback questionnaires** (including website user experience, preparedness, communication)

##### Timepoint 1

Data will be collected at two time points. Firstly, environmental scan interviews will be conducted with nursing home managers, staff and family members in each participating home. The aim of the environmental scan is to provide a facility profile describing the relevant characteristics of the nursing home, current practice and to identify potential barriers and facilitators for accessing and implementing learning. A nursing home profile questionnaire will be administered to nursing home managers. The environmental scan provides valuable context for the case study analysis.

Interviews will take approximately 30–60 min. Once the first interview is complete, participants will be emailed details on how to access the training and information resources (website). After care staff and family members have accessed and navigated to the end of the online resources, they will be required to complete an automated online evaluation questionnaire.

##### Time point 2: 1–2 weeks post intervention

A follow up interview with all participants in timepoint 1 will be conducted 1–2 weeks after participants have accessed the training or information resources. All interviews will be conducted by telephone or on MS Teams. Interviews will take approximately 30–60 min.

#### Development of qualitative data collection tools

The Technology Assessment Model (TAM) framework will be used to develop the interview guides with nursing home staff and family members. This is a conceptual framework based on the Theory of Reasoned Action (TRA) first developed by Fishbein and Ajzen [17]. It is a social psychology model for explaining human behaviour that posits the individuals will undertake an action when they possess a positive attitude toward performing the action (behavioural beliefs) and when they perceive others think they should perform it (normative beliefs). Davis [18] developed the TAM from TRA to predict and explain acceptance and evaluations of IT systems. More specifically, TAM highlights two beliefs as predicting acceptance and evaluations of IT systems – the extent to which a system is perceived as easy to use and how useful it will be in their job role. TAM has been frequently used by researchers evaluating e-learning, including developing qualitative research tools [19].

### Recruitment of nursing homes

We will purposively recruit 6–9 nursing homes in Northern Ireland, Scotland and England based on characteristics and profiles such as; location, independent, private and or part of a group/chain; residents with a range of needs (e.g. not just older adults). The final sample will include nursing homes with different profiles. Residential care homes and facilities not offering nursing care to their residents will be excluded from this study.

### Recruitment of nursing home staff

Nursing home managers of sites that match our criteria will be approached and invited to participate in the study. The nursing home manager of each of the eligible participating nursing homes will then identify other eligible nursing home staff (i.e. registered nurse or health care assistant) at each facility who meet the inclusion criteria and either post/email them an information pack to include a letter of invitation, Participant Information Sheet (PIS), consent form and response slip. They will be asked to complete the response slip and consent form and return them to the research team in either the pre-paid envelope provided or via email, if they are interested in taking part in the research. The researchers contact details are on the PIS should the nursing home staff need any further information prior to agreeing to participate. One to 2 weeks after the information packs have been given out, the nursing home manager will remind staff about the research and that if they are interested to follow up with the researcher or post back/email the response slip and consent form within one to 2 weeks.

For those nursing home staff who return a response slip and consent form indicating they are happy to participate in the research, the research team will follow up with them, answer any questions and if they are still happy to continue, arrange the interviews.

### Inclusion/exclusion criteria for nursing home staff

Inclusion criteria

- Registered nurse with the Nursing and Midwifery Council or health care assistant

### Recruitment of family members

The nursing home manager(s) will identify eligible family members, provide a brief introduction to the study and ask for their consent to be contacted by a researcher. If consent is given, a researcher will make contact and provide an overview of the study, answer any immediate questions and email or post them an information pack (to include a letter of invitation, Participant Information Sheet (PIS) and consent form). The researcher’s contact details will be on the PIS should the family member need any further information prior to agreeing to participate.

One to 2 weeks after the information pack has been sent out, non-responders will be followed up by the researcher who will send a second letter of invitation either by post or email asking them to respond within one to 2 weeks.

For those family members who have returned the consent form indicating they are happy to participate, a researcher will follow up with them and answer any questions. If they are still happy to continue, the researcher will organise the interview at a convenient date and time.

### Inclusion/exclusion criteria for family members

Inclusion criteria

- Aged 18 years or older
- Be the individual most involved in the care of the resident as identified by the nursing home manager
- Can understand written and verbal English
- Have access to digital devices e.g. mobile phone, tablet, laptop or desk top computer.

Exclusion criteria

- Aged < 18 years
- Family members who are unable to communicate through written and spoken English

### Consent process

All staff and family member participants will provide informed consent. The researchers will seek written consent, but if a participant is unable to receive/return consent by mail or email, verbal consent will be audio recorded as a last resort. Participants will be given at least 48 h to consider taking part in the research from receipt of the PIS and consent form and will have the opportunity to discuss the research and ask questions. During all aspects of the research the researcher will use process consent whereby they will regularly evaluate the comfort of the participant, and if or where appropriate, offer them the option to decline to answer specific questions or terminate their involvement at any time.

If any of the participants become upset, a distress protocol will be followed and support packs will be made available if required to both nursing home staff and family members.

### Data management and analysis

Within each nursing home, data will be analysed 1) within data set (nursing home), followed by a 2) cross-case analysis. The analysis will be guided by the RE-AIM framework [16]. Reach will be assessed by website analytics (numbers of visits, time spent on site/sections; pathway through website). The domains; Effectiveness, Adoption, Implementation, and Maintenance, will be assessed through quantitative descriptive indicators and qualitative interviews with both nursing home staff and family members.

Interviews will be digitally recorded and transcribed verbatim. We will analyse qualitative data in NVIVO and follow the framework analysis style outlined by Ritchie and Spencer [20]. Descriptive statistics will be used to analyse the quantitative data. Syntheses across quantitative and qualitative data will occur at the interpretation stage creating a single nursing home case study. The nursing home case study will be summarized in a logic model. Following individual nursing home case study analysis, a cross case synthesis will be performed across all case study sites. Following the evaluation and ERG consultation, the online training and information resources will be refined and implementation guidelines developed.

### Ethical and governance issues

In order to minimise participant burden, the total duration of interviews will be limited to a maximum of 60 min. Interviews will be undertaken by experienced researchers in the field of nursing home research.

Participation will incur time and some interviews may cover emotional and distressing issues. Participation is voluntary and any participant can choose to end their involvement in the study without needing to give a reason why. However, we also anticipate several benefits from taking part in this study. It is anticipated that nursing home staff and family members will be more knowledgeable regarding end-of-life options for residents with COVID-19 and feel more equipped in having ACP conversations.

Involvement in the study will be kept confidential. Participant information will only be accessible to members of the research team. Personal information will be coded with a unique identifier number (a number linked to participants name which only the research team will have access to on an encrypted file). We will keep all information safe and secure on Microsoft Teams (GDPR compliant), and participants’ identities will be anonymised in any publications or other outputs. Digital recordings will be deleted as soon as the transcript is transcribed. All information will be treated as strictly confidential and handled in accordance with the Data Protection Act 2018.

### Dissemination

It is envisaged that the study will produce a number of outputs, including: news media; digital media targeted at different stakeholders (such as family members, nursing home staff, policy makers, researchers and academics); conference presentations and webinars; open access publications in academic journals. The training and information resources for nursing home staff and family members will also be disseminated and maintained online. Dissemination plans concerning the training and information resources will be finalised based on discussions within the research team, ERG and IOP.

## Discussion

### Strengths

This study is very timely given the challenging experiences of nursing homes, their staff, residents and family members during the COVID-19 pandemic. The protocol itself is an example of how research can be adapted to take place during a pandemic in very challenging settings and circumstances.

When compared to existing research in the field of COVID-19 and ACP, this study is innovative and ambitious in scale. An existing review of ACP in the context of COVID-19 [3] only found one small pilot study that evaluated an ACP tool during the COVID-19 pandemic. The tool was piloted on 17 older adults in residential care in the United States, and only staff were involved in the evaluation [21]. On this basis, in developing online ACP training resources for nursing home staff and information resources for family members, and involving both groups in evaluation, this study meets a key need and addresses an important gap in research. The use of a multiple case study design will evaluate the training and information resources in their social and organisational context and during the context of the COVID-19 pandemic, and we anticipate collecting some rich and in-depth data.

While it is hoped that the training resources and information resources will impact on policy and practice in the nursing homes that are recruited to the study, the development of implementation guidelines will ensure impact on wider national policy and practice. We will also work with the ERG and IOP to attain this impact. The resources will be ‘COVID-centric’, and the collaboration with the integrated communications company will ensure that the online training and information resources will be visually appealing, easily accessible and professionally filmed. This will ensure high quality content that will be able to be updated. The resources will be sustainable beyond the duration of the study and this will enable the resources to have a longstanding relevance for future ACP practice.

### Limitations and challenges

Although it is clearly good practice to involve residents in developing advance care plans where possible, accessing nursing homes in order to *meaningfully* include residents as a participant group at the time of the grant award and study design was deemed too problematic due to the ongoing COVID-19 pandemic. In addition to not being able to gain physical access to nursing home sites, on account of social distancing / infection control restrictions, another factor was the relatively short period of time between the award of the grant and expiration of the funding. However, resident involvement will be promoted in the online training resources.

Future research could focus on supporting nursing home staff, residents and family members to enact ACP discussions based on engaging with the training and information resources. The online nature of the training and information resources that we will develop in this study has been driven by the need to offer materials in the context of social distancing restrictions. While this will enable nursing home staff and family members to access the resources at a time of their choosing, it is not within the scope of this project to offer support face-to-face or in-person. As such one limitation in this study is that we are not evaluating or making comparisons with offline methods of providing training or information.

There is a paucity of high-quality research conducted in nursing homes, particularly in the pressing area of the effectiveness of staff training and information resources for family members on ACP in nursing homes during the COVID-19 pandemic. This work will stimulate further innovative inquiry for staff education in nursing homes. The field of information resources for family members of nursing home residents is arguably even less developed, and as such this study will make an important contribution this area.

## Data Availability

De-identifiable data and study materials will be shared and requests will be judged on an individual basis.

## Abbreviations

ACP: Advance Care Planning
ERG: Expert Reference Group
IOP: International Observer Panel
RE-AIM: Reach, Effectiveness, Adoption, Implementation, Maintenance Framework
TAM: Technology Assessment Model
TRA: Theory of Reasoned Action
TRIP: Turning Research Into Practice database
